# Motivational modulation of bradykinesia in Parkinson’s disease off and on dopaminergic medication

**DOI:** 10.1007/s00415-014-7315-x

**Published:** 2014-04-01

**Authors:** Maja Kojovic, Pablo Mir, Iris Trender-Gerhard, Susanne A. Schneider, Isabel Pareés, Mark J. Edwards, Kailash P. Bhatia, Marjan Jahanshahi

**Affiliations:** 1 Sobell Department of Motor Neuroscience and Movement Disorders, UCL Institute of Neurology, The National Hospital for Neurology and Neurosurgery, 33 Queen Square, London, WC1N 3BG UK; 2 Department of Neurology, University of Ljubljana, Ljubljana, Slovenia; 3 Unidad de Trastornos del Movimiento, Servicio de Neurología y Neurofisiología Clínica, Instituto de Biomedicina de Sevilla, Hospital Universitario Virgen del Rocío/CSIC/Universidad de Sevilla, CIBERNED, Seville, Spain; 4 Genetic Medicine Department, University of Manchester, Manchester, UK; 5 Department of Neurology, University of Kiel, Kiel, Germany

**Keywords:** Parkinson’s disease, Bradykinesia, Motivation, Reward, Reaction time

## Abstract

Motivational influence on bradykinesia in Parkinson’s disease may be observed in situations of emotional and physical stress, a phenomenon known as paradoxical kinesis. However, little is known about motivational modulation of movement speed beyond these extreme circumstances. In particular, it is not known if motivational factors affect movement speed by improving movement preparation/initiation or execution (or both) and how this effect relates to the patients’ medication state. In the present study, we tested if provision of motivational incentive through monetary reward would speed-up movement initiation and/or execution in Parkinson’s disease patients and if this effect depended on dopaminergic medication. We studied the effect of monetary incentive on simple reaction time in 11 Parkinson’s disease patients both “off” and “on” dopaminergic medication and in 11 healthy participants. The simple reaction time task was performed across unrewarded and rewarded blocks. The initiation time and movement time were quantified separately. Anticipation errors and long responses were also recorded. The prospect of reward improved initiation times in Parkinson’s disease patients both “off” and “on” dopaminergic medication, to a similar extent as in healthy participants. However, for “off” medication, this improvement was associated with increased frequency of anticipation errors, which were eliminated by dopamine replacement. Dopamine replacement had an additional, albeit small effect, on reward-related improvement of movement execution. Motivational strategies are helpful in overcoming bradykinesia in Parkinson’s disease. Motivational factors may have a greater effect on bradykinesia when patients are “on” medication, as dopamine appears to be required for overcoming speed-accuracy trade-off and for improvement of movement execution. Thus, medication status should be an important consideration in movement rehabilitation programmes for patients with Parkinson’s disease.

**Electronic supplementary material:**

The online version of this article (doi:10.1007/s00415-014-7315-x) contains supplementary material, which is available to authorized users.

## Introduction

Motivational influence on movement speed is evident in temporally pressing situations. Common examples include working faster when facing a deadline, walking faster when late for an appointment or achieving personal bests in sports. Experimental evidence implies that self-determined “highest” speed of movement is set below the possible maximum, allowing for an increase of movement speed in the presence of external challenges or motivational factors [1–3].

Motivational modulation of movement speed is important in understanding variability of bradykinesia in Parkinson’s disease (PD) including paradoxical kinesis, which refers to unique episodes of sudden and brief improvements of mobility in situations of emotional or physical stress [4, 5]. PD patients also enhance their motor performance in response to appropriate auditory, visual or tactile cues [6, 7] and can benefit from attentional strategies [8], suggesting that various factors may improve bradykinesia, possibly through similar mechanisms.

The locus of the effect of motivational influences on movement speed in PD remains unclear, and it is not known which specific aspects of movement are affected by provision of motivational incentive. In particular, it is not known if manipulation of motivation improves movement preparation/initiation or execution (or both) and if this effect depends on the patients’ medication state. One way to experimentally test motivational influences on movement initiation and execution is through reaction time and reward studies. We have previously shown in healthy participants that monetary reward is a relevant incentive that speeds up reaction times [9]. Here, we tested whether the prospect of monetary incentive affects initiation time (IT) and movement time (MT) in PD patients and if this effect depends on the patients’ medication state. We evaluated PD patients “on” and “off” dopaminergic treatment and compared them to healthy participants.

## Methods

### Participants

We studied 11 patients with idiopathic PD diagnosed according to UK Brain Bank Criteria [10], without significant tremor or dyskinesias (7 men, 4 women, mean age 62, range 50–70) and 11 aged-matched healthy participants (5 men, 6 women, mean age 61, range 51–70). Patients were recruited from the PD outpatient clinic at the National Hospital for Neurology and Neurosurgery in Queen Square, London. None of the patients had pathological gambling or other impulse control disorders, as assessed by the question on dopamine dysregulation syndrome of the MDS-UPDRS scale (Question 1.6). The clinical and demographic characteristics of the patients are given in Table 1. Only patients without previous clinical diagnosis of depression, apathy or cognitive impairment were included. Healthy participants were recruited from the list of healthy participants maintained by the Cognitive Motor Neuroscience Group of the Sobell Department of Motor Neuroscience and Movement Disorders. They were selected on the basis of good general health, and none had a history of neurological or psychiatric illness, head injury or drug or alcohol misuse. According to the Edinburgh Handedness Inventory [11] participants were strongly right-handed (PD patients: mean 85.9, SEM 0.4; Healthy Participants mean 88.2, SEM 0.32). Written informed consent was obtained from all participants and the study was approved by the local ethics committee and conducted in accordance with the Declaration of Helsinki.

**Table 1:** Parkinsons’s disease patients-demographic and clinical characteristics

Sex	Age	Disease duration	More affected side	H&Y	UPDRS off	UPDRS on	MMSE	MAS off		MAS on		BDI off		BDI on		TPQ-ns	TPQ-ha	TPQ-rd	Drugs	LED (mg)
M	50	8	R	3	28	15	30	15	11		5		7		4		17	9	Rop	120
M	62	3	L	3	29	14	29	8	9		13		13		17		15	17	L-Dopa Ras	400
F	69	3	L	3	37	32	30	22	23		14		13		8		10	16	Rop L-Dopa	460
M	57	2	R	2	20	14	29	29	35		19		15		19		11	8	L-Dopa	300
M	62	5	R	3	49	20	30	16	5		10		3		17		18	18	Rop L-Dopa	620
F	57	3	L	2	15	4	30	7	5		4		7		13		8	12	Rop	200
M	70	8	R	2	26	15	30	17	17		10		19		19		18	17	L-Dopa	375
M	62	5	L	2	28	10	30	12	13		16		17		17		24	15	Pram L-Dopa	400
F	56	6	L	3	30	17	29	5	6		14		12		13		8	12	Pram L-Dopa	400
M	70	5	R	3	42	12	28	10	8		6		6		12		7	15	L-Dopa	375
F	68	6	R	3	40	9	30	16	12		5		4		16		8	15	L-Dopa	460
Average	62.1	4.9		2.6	31.3	14.7	29.5	14.7	13.7		10.1		10		14.1		13.1	14		373.7
SEM	2.0	0.6		0.15	0.15	2.15	0.2	2	2.6		1.5		1.6		1.4		1.7	1		40

*M* male, *F* female, *H&Y* Hoehn and Yahr stage, Motor section of *UPDRS* United Parkinson Disease Rating Scale (OFF, off medication; ON, on medication), *MMSE* Mini Mental State Examination, *MAS* Marin Apathy Scale, *BDI* Beck Depression Inventory, *TPQ* Tridimensional Personality Questionnaire, *ns* novelty seeking, *ha* harm avoidance, *rd* reward dependence, *Ras* rasegeline, *Rop* ropinirole, *Pram* pramipexole, *LED* L-Dopa Equivalent Dose in milligrams, *SEM* Standard Error of the Mean

### Experimental design

PD patients were studied in two different sessions separated by at least 1 week: in the practically defined “off” state after overnight withdrawal of medication and in the “on” state, approximately 1 h after their first morning dose of the usual medication and once clinical benefit was fully documented by neurological examination. The order of “off” and “on” sessions was balanced, with five patients being tested first in the “off” and six in the “on” state. Healthy participants completed one experimental session only.

### Experimental task: warned and unwarned simple reaction time

**Fig. 1:**
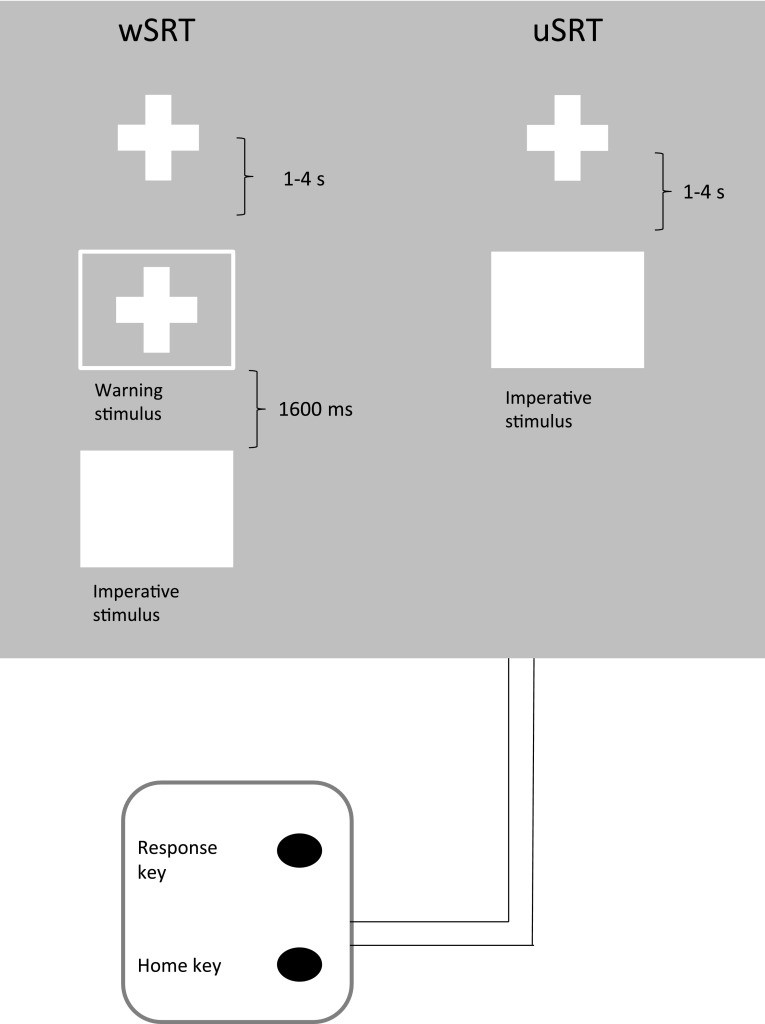
Illustration of the simple reaction time task, showing events presented on the computer screen at different stages of the unwarned and warned trials

Stimuli were presented on a computer screen and responses were made on a response box with two buttons: a home key and a response key (Fig. 1). Two buttons were 2.54 cm in diameter and placed in a vertical row, spaced 10.16 cm apart. In each session, participants completed 4 blocks of 100 trials each. Each block consisted of 50 warned simple reaction times (wSRT) and 50 unwarned simple reaction times (uSRT) trials randomly mixed. The participants sat in front of the computer monitor and were instructed to hold down the home key with the index finger of their right hand. On pressing the home key a fixation cross appeared on the screen. On wSRT trials, after a variable delay of 1–4 s, a warning signal (S1) was presented in the form of an empty square superimposed on the fixation cross. One thousand six hundred milliseconds later this square was filled to become solid white, and this constituted the imperative signal (S2). For wSRT trials, the participants were instructed to make use of the warning signal and prepare themselves to respond to presentation of the imperative signal. On presentation of the imperative signal, they were required to release the home key as quickly as possible and press the response key. The screen cleared 500 ms after a response was made. Participants then moved back to the home key in their own time. The next trial started when the home key was pressed again. On unwarned trials (uSRT), there was no S1 or warning signal and the fixation cross was followed, after a variable delay of 1–4 s, by a filled square (imperative stimulus), in which case the participant was required to move to and press the response key straight away. To discourage anticipatory responses, they were clearly instructed to wait for presentation of the imperative stimulus. There were five practice trials to familiarize participants with the stimulus presentation and the response of lifting the index finger from the ‘home’ key and moving to the ‘response’ key.

### Provision of monetary incentive for speeding up reaction times

The blocks were always organised in the same order (two unrewarded, followed by two rewarded), necessitated by the need to establish a RT baseline for each participant before introduction of monetary incentive. First two blocks were performed without financial incentive, with any changes in IT and MT from the first to second block providing a control for changes in ITs and MTs simply as a result of practice/task repetition effects. Participants were not told in advance that they would receive any reward in further blocks. At the end of the second block, participants were provided with feedback on their reaction time in the previous block (as an average of IT for uSRT and wSRT task) and were instructed that, for every 10 ms they speeded their reaction time in the third block, they would receive a monetary reward of 50 pence. At the end of the third block, they were again given feedback on their performance and whether they had speeded up relative to the previous block or not. The amount of money gained was displayed on the screen. Prior to the fourth block, participants were instructed that they would receive a monetary reward of 100 pence for every 5 ms they improved their reaction times in the following block. The monetary incentive was real and the participants were provided with the money gained at the end of the study. Using the same task without monetary incentive, we have previously shown that no significant practice effects occur from block 1 to block 4 in healthy participants [9].

### Measurement of initiation times, movement times and errors

The time from presentation of the imperative stimulus to the release of the ‘home’ key was measured as IT. The time from release of the ‘home’ key to pressing the ‘response’ key was measured as MT. Both IT and MT were recorded by the computer to the nearest millisecond. Two types of error were recorded: anticipation errors (IT less than or equal to 100 ms) and long responses (IT greater than 2,000 ms). Trials with error data were excluded and replaced with a new trial.

### Clinical and other measures

Severity of motor symptoms was assessed with the motor examination sections of the Unified Parkinson’s Disease Rating Scale (MDS-UPDRS) [12]. To screen for depression, apathy and cognitive impairment, we used the Beck Depression Inventory (BDI), Marin Apathy Scale (MAS) and Mini Mental State Examination (MMSE), respectively [13–15]. The Tridimensional Personality Questionnaire (TPQ) [16] was used to assess responsiveness to reward, harm avoidance and novelty seeking dimensions of personality. PD patients completed the MMSE and TPQ only once, while they were assessed both “off” and “on” medication for UPDRS, BDI and MAS.

### Statistical analysis

Monetary incentive may affect movement initiation and movement execution differently; therefore, the effect of monetary incentive on IT and MT was analysed separately. Since all patients performed the task with their dominant hand, which in six patients was the more affected and in five patients the clinically less affected side (even though all patients had bilateral symptoms at the time of the study, as evident from the Hoehn and Yahr stage), we first addressed if the asymmetry of the motor symptoms could have affected task performance. We conducted a preliminary ANOVA with the PD GROUP (two levels: patients with the dominant side more affected and patients with dominant side less affected) as the between-subject factor, and the within- subjects factors MEDICATION STATE (“on” vs. “off”), TASK (wSRT vs. uSRT) and BLOCK (1–4). We then compared all PD patients “on” and “off” medication in ANOVAs with factors MEDICATION STATE (“on” vs. “off”), TASK (wSRT vs. uSRT) and BLOCK (1–4) as within-subject factors. As a secondary analysis, we assessed how IT and MT compared between PD patients and healthy participants: data from PD patients “on” and “off” medication were separately compared to healthy participants in ANOVAs with the factor Group (PD “off” vs. healthy participants or PD “on” vs. healthy participants) as a between-subject factor, and factors TASK and BLOCK as within-subject factors. Post-hoc Tukey tests with corrections for multiple comparisons were used to further analyse significant main effects or interactions. Group differences in anticipation errors and long responses were analysed with a non-parametric Kruskal–Wallis ANOVA, followed by Wilcoxon signed rank test or Mann–Whitney *U* tests. Since clinical scales are ordinal scales, we compared differences between PD patients “off” and “on” medication or between patients and healthy participants, using non-parametric tests. The significance level was set at *p* ≤ 0.05.

## Results

The scores for the PD patients on UPDRS, MMSE, BDI, MAS, and TPQ are given in Table 1. For healthy participants, the means and standard error of the means were: BDI 5.81 (1.6); MAS 12.2 (1.53); MMSE 29.5 (0.4); TPQ novelty seeking 14.1 (1.7); TPQ harm avoidance 13 (2.1); and TPQ reward dependence 15.1 (1.2). As expected, in PD patients total motor UPDRS was higher in the “off” than the “on” state (*z* = −2.973: *p* = 0.003). There was no difference in BDI and MAS in PD patients “off” vs. “on” medication. We then averaged scores for BDI and MAS “off” and “on” and compared averaged values to those of healthy participants. PD patients scored worse than healthy participants on the BDI scale (*z* = −2.105; *p* = 0.03), while no difference was found for MAS, TPQ or MMSE.

The mean values and standard errors of IT and MT across blocks are shown in Supplementary Table 1.

### The effect of asymmetry of motor symptoms on IT and MT in PD patients

There were no differences between patients with the dominant side less vs. more clinically affected, as revealed by no significant effect of PD GROUP (IT: *F*(1,9) = 0.2; *p* = 0.66 and MT: *F*(1,9) = 0.31; *p* = 0.58), or PD GROUP × TASK or PD GROUP × MEDICATION STATE, PD GROUP × BLOCK or PD GROUP × BLOCK × MEDICATION STATE interactions. Thus, the clinical asymmetry of motor symptoms did not affect IT and MT and their change in response to reward or medication status.

### The effect of monetary incentive on IT and MT in PD patients “off” and “on” medication

**Fig. 2:**
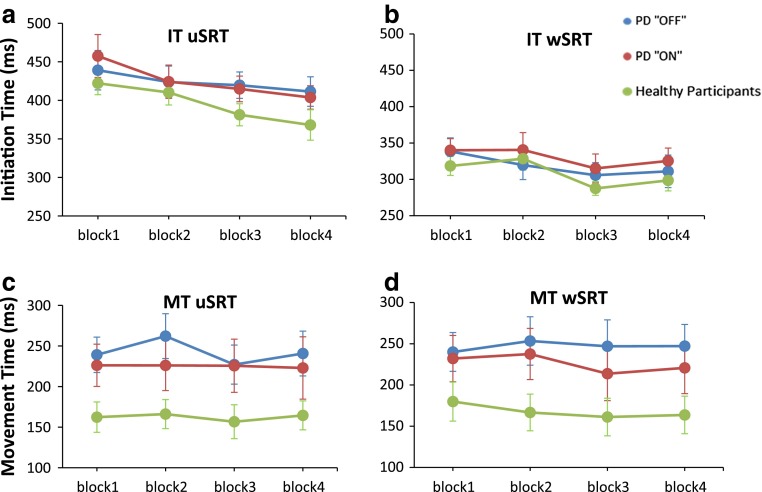
Initiation time (IT) and Movement time (MT) data for PD patients “off”and “on”medication and for healthy participants in the unwarned simple reaction time (uSRT) and warned simple reaction time (wSRT) task. Error bars are standard error of the mean. Data presented are the raw data. Blocks 1 and Block 2 are unrewarded blocks; Blocks 3 and Block 4 are rewarded blocks

Details of the statistical analysis are given in Table 2. For IT (Fig. 2a, b), the ANOVA revealed a significant main effect of BLOCK (*p* < 0.001), which was due to shorter IT in the rewarded Blocks 3 and 4 compared to the unrewarded Block 1 (*p* = 0.001 and *p* = 0.001, respectively), while there were no other differences between blocks (Block 1 vs. 2, *p* = 0.2; Block 2 vs. 3, *p* = 0.3; Block 2 vs.4, *p* = 0.2 and Block 3 vs. 4, *p* = 0.9). A 3-way interaction: MEDICATION STATE × TASK × BLOCK (*p* = 0.04) was significant, however, post hoc analysis revealed no differences or trend toward difference in IT between PD “off” and “on” in any of the four blocks, for either uSRT or wSRT. These results indicated that PD patients both “off” and “on” medication initiated their movement faster in blocks when the reward was provided.

**Table 2:** The results of the separate analyses of variance for initiation time (IT) and movement time (MT)

	IT		MT	
	*F* (*df*)	*p*	*F* (*df*)	*p*
PD “off” vs. PD “on” medication				
Medication state	0.63 (1,10)	0.4	0.57 (1,10)	0.5
Task	32.3 (1,10)	**<0.001**	0.32 (1,10)	0.6
Block	7.8 (3,30)	**<0.001**	1.9 (3,30)	0.1
Medication state × Task	0.69 (1,10)	0.4	0.14 (1,10)	0.7
Medication state × Block	0.31 (3,30)	0.81	0.36 (3,30)	0.8
Block × Task	0.91 (3,30)	0.4	0.03 (3,30)	1
Medication state × Task × Block	3.02 (3,30)	**0.04**	3.46 (3,30)	**0.03**
PD “off” medication vs. Healthy Participants				
Group	0.98 (1,20)	0.3	6.0 (1,20)	**0.02**
Task	58 (1,20)	**<0.001**	1,0 (1,20)	0.3
Block	14.7 (3,60)	**<0.001**	1.6 (3,60)	0.2
Group × Task	0.48 (1,20)	0.5	(1,20)	0.9
Group × Block	2.0 (3,60)	0.1	1,1 (3,60)	0.4
Block × Task	1.2 (3,60)	0.3	1.1 (3,60)	0.4
Group × Task × Block	0.82 (3,60)	0.5	1.14 (3,60)	0.4
PD “on” medication vs. Healthy Participants				
Group	1.6 (1,20)	0.2	2.7 (1,20)	0.1
Task	88.7 (1,20)	**<0.001**	0.4 (1,20)	0.5
Block	13.6 (3,60)	**<0.001**	1.2 (3,60)	0.3
Group × Task	0.15 (1,20)	0.7	0.23 (1,20)	0.6
Group × Block	0.75 (3,60)	0.5	0.15 (3,60)	0.9
Block × Task	3.73 (3,60)	**0.02**	1.6 (3,60)	0.2
Group × Task × Block	0.09 (3,60)	1	1.19 (3,60)	0.3

*PD* Parkinson’s disease

The significant effects are shown in bold type

For MT, the effect of monetary incentive was different between patients “off” and “on” medication (Fig. 2c, d), as indicated by a significant 3-way interaction MEDICATION STATE × TASK × BLOCK interaction (*F* (3, 30) = 3.46; *p* = 0.03), for which the post hoc analysis showed to be determined by differences in MT in uSRT and wSRT depending on medication status. For uSRT, PD “on” were faster than “off” in the unrewarded condition (Block 2; *p* = 0.01). Notably, in response to reward, PD patients had faster MT in “on” than in “off” state for the wSRT condition (Block 3; *p* = 0.02).

PD patients, irrespective of medication status, benefited from a warning signal to initiate and execute the movements faster, as revealed by a significant effect of TASK in the ANOVAs for both IT and MT (*p* < 0.001 and *p* < 0.001, respectively) (Table 2).

### The effect of monetary incentive on IT and MT in PD patients versus healthy participants

For IT, ANOVA (Table 2) confirmed the significant main effects of TASK and BLOCK noted above, but revealed no difference in reward responsiveness between PD patients (irrespective of treatment status) and healthy participants, as there were no significant GROUP X BLOCK or GROUP X TASK X BLOCK interactions. For PD “off” vs. healthy participants, the factor BLOCK was significant due to shorter ITs in blocks 3 compared to both blocks 2 and 1 (*p* < 0.001 and *p* = 0.003, respectively) and shorter IT in block 4 compared to block 1 and 2 (*p* < 0.001 and *p* = 0.001, respectively), while there was no other difference between blocks. Similarly, for PD “on” vs. healthy participants, the factor BLOCK was again significant due to shorter ITs in Blocks 3 compared to Block 2 and 1 (*p* < 0.001 and *p* = 0.002, respectively) and shorter IT in Block 4 compared to Block 1 and 2 (*p* < 0.001 and *p* = 0.002, respectively), while there was no other differences between blocks.

For MT, there was no difference in reward responsiveness between patients either “off” or “on” medication and healthy participants as indicated by no significant GROUP × BLOCK or GROUP × TASK × BLOCK interactions. PD “off” medication had significantly slower MTs than healthy participants across all blocks (main effect of GROUP* p* = 0.02).

### Anticipation errors and long responses

**Fig. 3:**
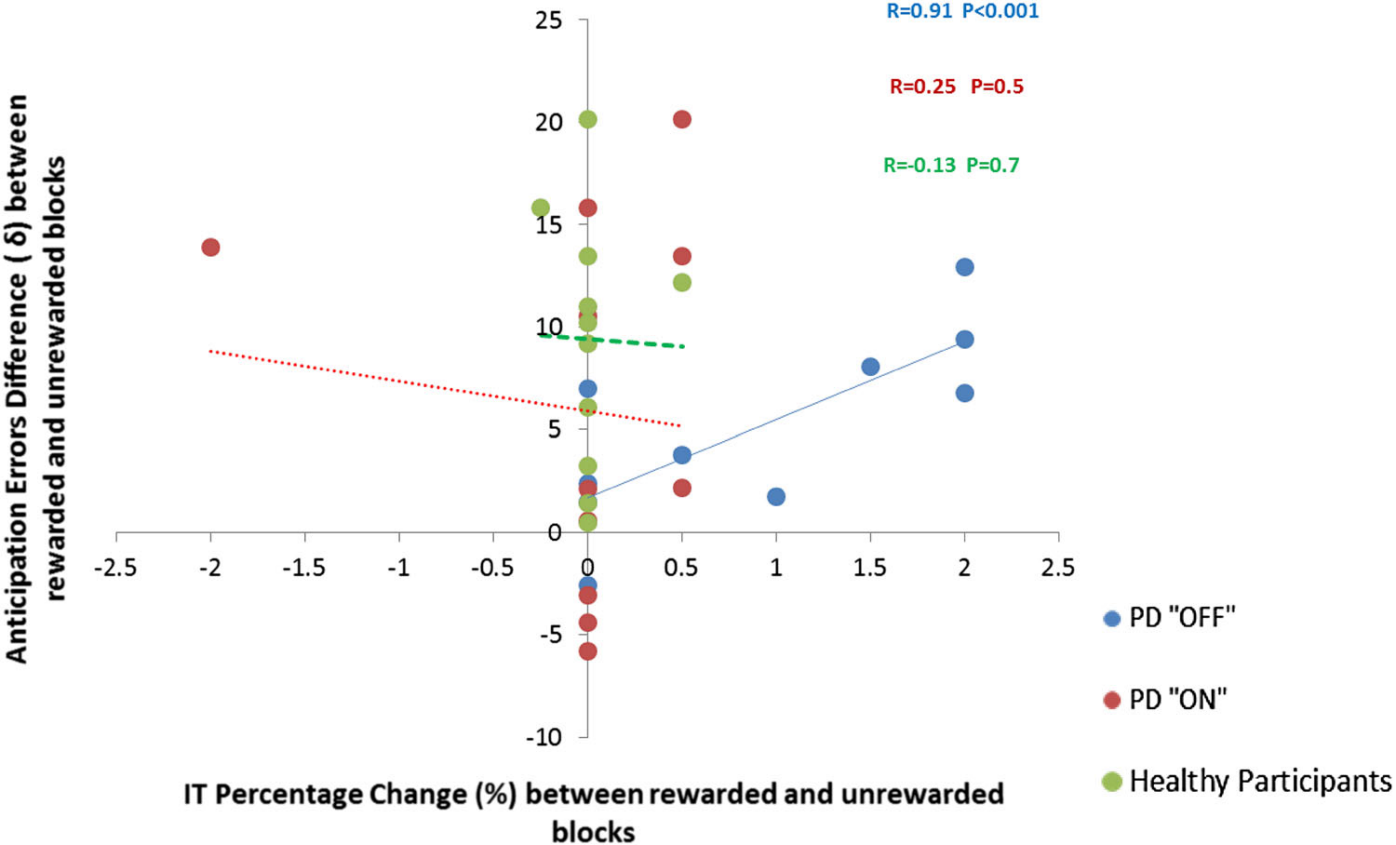
PD patients “off”medication showed a significant positive correlation between improvement of IT in response to prospect of monetary reward and the number of anticipation errors, suggestive of “speed-accuracy trade-off”. This association was not present in PD patients “on” medication or in healthy participants. Percentage of IT improvement is plotted on the *x*-axis and larger values correspond to greater improvement in IT in response to prospect of reward. Difference in anticipation errors between rewarded and unrewarded blocks is plotted on the y-axis and a larger value indicates more anticipation errors in rewarded trials

PD patients “off” had a significantly higher number of anticipation errors (AEs) than “on” medications. (*χ* ^2^ (2, *N* = 33) = 25.5; *p* < 0.001). This was due to more AE in rewarded Blocks 3 and 4 (*p* = 0.01 and *p* = 0.03, respectively) in “off” comparing to “on” medications, while no such difference in AE was present for unrewarded Blocks 1 and 2 (Supplementary file). Further, analysis revealed that when “off” medications, PD patients also made more AEs than healthy participants in the rewarded Blocks 3 and 4 (*p* = 0.01 and *p* = 0.03, respectively), but not in the unrewarded Blocks 1 and 2. When “on” medication, no such a difference in AE between PD and the healthy participants was seen. Importantly, PD patients “off” medications showed a strong positive correlation between the percentage of improvement of IT in rewarded blocks and the number of anticipation errors (*r* = 0.92; *p* < 0.001) (Fig. 3). There was no difference in the number of long responses between PD patients “off” and “on” medication or between PD patients and controls (median 0, range 0-3 across all four blocks).

None of the Spearman correlations between MAS, BDI, MMSE or TPQ and IT or MT were notable or significant for PD patients or healthy participants.

## Discussion

The main findings of the present study are: (1) The prospect of reward improved movement initiation in PD patients irrespective of their medication status and to a similar extent as in healthy participants. (2) For patients tested “off” medication, reward-related improvement of movement speed was associated with an increased frequency of anticipation errors. (3) Dopamine replacement eliminated the anticipation errors and also influenced the reward-related improvement of movement execution.

Our finding that even “off” medication patients improved their ITs with the prospect of monetary incentive (Fig. 2a,b) may perhaps be surprising, but is consistent with previous studies, which demonstrated a preserved ability of PD patients to translate the expectation of reward into generation of faster movements or greater physical force, even when unmedicated. For example, non-apathetic bradykinetic PD patients “off” medication, in the presence of monetary incentive, were able to increase hand grip force [17] or to complete a spatial search task faster [18]. Nevertheless, our results are different from those of Shiner et al. [19], who reported that PD patients “off” dopaminergic treatment failed to modulate movement speed in the face of monetary reward. However, since these authors considered only changes in MT, improvements in IT that we describe here, could have been missed.

There are several possible explanations for our finding that PD patients “off” dopaminergic treatment behave no differently than “on” treatment when the effect of monetary incentive on IT is concerned. First, it has been suggested that the neurodegenerative process in early PD selectively spares dopaminergic limbic circuits that mediate motivational influences on behaviour [20–22], while preferentially affecting the nigrostriatal dopaminergic projections responsible for bradykinesia. Indeed, circuits believed to mediate motivational influences on behaviour that originate from the anterior cingulate and orbitofrontal cortices and then synapse on the ventral striatum and ventral pallidum, remain relatively unaffected until the advanced stages of PD [23]. Second, an alternative explanation is engagement of compensatory circuits during reward-related tasks. PD patients “off” treatment could have made faster movements in the presence of monetary incentive by using dopamine-independent compensatory pathways. It has been found that compensatory cerebellar activation underpins motivational modulation of motor behaviour in non-medicated PD patients [1, 3, 18, 24, 25]. A compensatory role of cerebellar circuitry has also been hypothesized by Keefe et al. [26] and Glickstein and Stein [27] in paradoxical kinesis and demonstrated in imaging studies of motor urgency in PD [20, 24]. Third, our results could also be considered within the theoretical framework that proposes dual, goal-directed and habitual systems of movement control [28]. Studies in animals and humans have identified spatially segregated functional territories in the basal ganglia for the control of goal-directed and habitual actions [29, 30]. In PD, loss of dopamine is predominantly in the posterior putamen [31], a region of the basal ganglia, which is preferentially activated in situations when habitual control of movement is implicated. The prospect of reward could have switched motor control from habitual to a goal-directed mode, mediated by the comparatively preserved processing in the rostromedial striatum, thus, resulting in faster movements.

It has been suggested that the motor system has its own motivation circuit, which operates implicitly (i.e., outside awareness) to direct behaviour [19, 32]. The proposal is that movement speed is determined implicitly by a value assigned to the goal of the movement. How fast one moves will depend on the optimal balance between the “cost” of movement, i.e., time and effort necessary to complete the action, and the reward obtainable in the particular behavioural setting [5, 32]. According to this model, bradykinesia in PD is a result of a shift in the cost/benefit ratio towards slower movements,caused by dopaminergic deficit. PD patients might find it more of an effort to move fast and would implicitly prefer slower movements [5], despite the repertoire of normal movements being preserved, as evidenced by improvements in movement speed in response to different types of cueing, reward or in stressful situations [20, 32, 33]. Accordingly, a provision of explicit monetary incentive in our experiment could override the implicit energetic cost bias hypothesised by Mazzoni et al. [32] and thus change the cost/benefit ratio toward faster movements.

A novel finding of the present study is that certain aspects of the effect of motivation on movement speed (induced by the prospect of a monetary incentive) were differently modulated in PD patients depending on their medication status. In our patients, provision of monetary incentive differentially affected movement execution depending on dopaminergic status, as only patients “on” medication improved MT in response to a prospect of reward (Fig. 2d). Dopamine neurotransmission is known to exert a powerful influence over movement vigour, that is the likelihood of moving at a certain speed or strength; and motivational impact on movement depends on tonic dopamine release in the nigrostriatal circuits [33–35]. We, thus, speculate that the medication effect on MT may have been mediated through dopaminergic modulation of movement vigour. Indeed, a recent study using a temporal decision making task also showed that a tendency to adapt response times to achieve maximal reward depends on patients’ medication status [36]. In addition, in PD patients “off” treatment improvement of ITs in response to prospect of monetary reward was associated with increased frequency of anticipation errors, which was then eliminated in the “on” state (Fig. 3). Therefore, our results suggest that dopamine was required for avoiding speed-accuracy trade-offs during movement execution, by preventing premature responses.

Finally, in the overall interpretation of our results we should consider also the effect of attention. It is known that focusing attention on movement effect (i.e., goal of the movement) rather than focusing on movement itself (i.e., execution of movement) enhance motor performance and improves both movement speed and accuracy of movement [37, 38]. Levodopa or dopamine agonists improve attention span and vigilance [36] and attentional strategies help to improve motor speed and accuracy of movement in PD patients “on” medication [32]. Thus, improved focusing of attention with prospect of reward could have acted synergistically with dopamine replacement and the presence of temporal cueing in the wSRT task [36], resulting not only in improvement of MT, but also (by keeping premature responses in check and preventing anticipatory errors) in overall more accurate performance in the “on” state.

Similar to our previous investigation in young healthy participants [9], in the present study the reward magnitude had no significant effect on speeding up RTs, as there was no further improvement either in movement initiation or execution times in response to a higher level of reward. This is likely due to a “ceiling” effect, as participants could have already exceeded their self-determined maximal movement speed with the first level of the reward.

A limitation of our study was that the blocks were always organised in the same order (two unrewarded, followed by two rewarded), dictated by the need to establish a RT baseline for each participant before introduction of monetary incentive. We cannot exclude that the improvement in rewarded blocks was affected by practice. However, this seems unlikely, because previous studies showed no evidence of such practice effects across repeated blocks in PD patients or in healthy participants [9, 19, 39]. Furthermore, we observed no significant speeding of RTs from the unrewarded Block 1 to Block 2. Another potential limitation is that PD patients completed the task twice (“off” and “on” medication) and therefore were aware of the experimental design when they performed the task for the second time. However, as the order of “off” and “on” sessions was counterbalanced across patients, we believe this potential confounding effect was minimized and did not influence the results. In future studies, a subjective rating of the value of the monetary reward to participants would help establish its motivational and incentivizing impact in each case.

## Conclusions

Slowness of movement in PD may be improved in exceptional situations that trigger paradoxical kinesis [4, 5] or when external stimuli for the guidance of movement are provided 8, 40]. Here, we show that motivational processes, over and above emotional influences or sensory cueing, may challenge and overcome bradykinesia in PD. This motivational modulation of movement speed seems to be more efficient during the medicated (“on”) state, which may have implications for choosing the most appropriate conditions for rehabilitation of PD patients.

## Electronic supplementary material

Below is the link to the electronic supplementary material.

Supplementary material 1 (DOCX 16 kb)

Supplementary material 2 (DOCX 23 kb)
