# Supplementary material for: Motivational modulation of bradykinesia in Parkinson’s disease off and on dopaminergic medication
Source: J Neurol. 2014 Apr 1;261(6):1080–9. doi: 10.1007/s00415-014-7315-x (PMC4057625; doi:10.1007/s00415-014-7315-x)
Supplement: Supplementary file 1 — Supplementary material 1 (DOCX 16 kb) [file 415_2014_7315_MOESM1_ESM.docx]

**SUPPLEMENTARY FILE**

There was no difference in the number of long responses (LR) between PD patients “off” and “on” medication or between PD patients and controls (**Supplementary table 2**). When tested “off” medication, PD patients had a significantly higher number of anticipation errors (AEs) (χ^2^ (2, *N* = 33) = 25.5; p<0.001) compared to “on” state. This was due to more AEs in PD “off” compared to “on” medication in rewarded blocks (block 3: PD “off” median:2, range 0-4, PD “on” median 0, range 0-1; z=-2.55; p= 0.01; block 4: PD “off” median 0, range 0-3, PD “on” median 0, range 0-2; z=-2.33; p=0.03, Wilcoxon sign ranked test). There was no differences in AEs between PD patients “off” and “on” in unrewarded blocks (block 1: PD “off” median 0, range 0-2, PD “on” median 0, range 0-3; block 2: PD “off” median 0, range 0-2, PD “on” median 0, range 0-2). Similarly, PD “off” made more AEs than healthy participants in the rewarded blocks (z=-2.65 p= 0.01 for block 3 and z=-2.19; p=0.03 for block 4, Mann-Whitney U test), but not in the unrewarded blocks 1 and 2. For the healthy participants the median number of AEs across the 4 blocks was 0, with a range of 0-1. There was no difference in AE between PD “on” and the healthy participants.

|  | | **BLOCK 1** | | **BLOCK 2** | | **BLOCK 3** | | **BLOCK 4** | |
| --- | --- | --- | --- | --- | --- | --- | --- | --- | --- |
|  |  | **AE** | **LR** | **AE** | **LR** | **AE** | **LR** | **AE** | **LR** |
| **PD »OFF«** | MEDIAN  INTERVAL | 0  0-2 | 0  0-1 | 0  0-2 | 0  0-0 | 2  0-4 | 0  0-1 | 0  0-3 | 0  0-2 |
| **PD«ON«** | MEDIAN  INTERVAL | 0  0-3 | 0  0-3 | 0  0-2 | 0  0-0 | 0  0-1 | 0  0-1 | 0  0-2 | 0  0-0 |
| **HEALTHY PARTICIPANTS** | MEDIAN  INTERVAL | 0  0-1 | 0  0-0 | 0  0-0 | 0  0-1 | 0  0-1 | 0  0-1 | 0  0-1 | 0  0-1 |

Supplementary table 2: The number of anticipation errors and long responses

Abbreviations: AE, anticipation errors; LR, long responses
