# Supplementary material for: Motivational modulation of bradykinesia in Parkinson’s disease off and on dopaminergic medication
Source: J Neurol. 2014 Apr 1;261(6):1080–9. doi: 10.1007/s00415-014-7315-x (PMC4057625; doi:10.1007/s00415-014-7315-x)
Supplement: Supplementary file 2 — Supplementary material 2 (DOCX 23 kb) [file 415_2014_7315_MOESM2_ESM.docx]

|  | | Block 1 | | Block 2 | | Block 3 | | Block 4 | |
| --- | --- | --- | --- | --- | --- | --- | --- | --- | --- |
|  | | IT | MT | IT | MT | IT | MT | IT | MT |
| PD OFF | uSRT  wSRT | 439(25)  338(19) | 239(21)  240(23) | 423(21)  319(20) | 254(27)  253(29) | 419(17)  305(17) | 227(24)  247(32) | 411(19)  311(22) | 240(27)  246(36) |
| PD ON | uSRT  wSRT | 457(28)  340(15) | 226(26)  232(28) | 424(22)  340(23) | 226(31)  237(31) | 414(17)  315(19) | 225(33)  213(32) | 403(15)  325(18) | 222(38)  220(31) |
| Healthy  Controls | uSRT  wSRT | 422(15)  318(13) | 162(19)  179(27) | 410(16)  328(16) | 166(18)  166(22) | 381(14)  287(10) | 156(21)  161(23) | 368(14)  298(13) | 164(18)  163(22) |

**Supplementary Table 1:** Mean and standard errors for Initiation times (IT) and movement times (MT) across 4 blocks

Values represent mean with standard error (SE).
